# Advanced glycation end products promote ROS production via PKC/p47 phox axis in skeletal muscle cells

**DOI:** 10.1186/s12576-024-00944-1

**Published:** 2024-10-05

**Authors:** Shinichiro Suzuki, Tatsuya Hayashi, Tatsuro Egawa

**Affiliations:** 1 Laboratory of Molecular Adaptations to Exercise, Graduate School of Human and Environmental Studies, Kyoto University, Yoshida-Nihonmatsu-Cho, Sakyo-Ku, Kyoto, 606-8501 Japan; 2 Laboratory of Sports and Exercise Medicine, Graduate School of Human and Environmental Studies, Kyoto University, Yoshida-Nihonmatsu-Cho, Sakyo-Ku, Kyoto, 606-8501 Japan

**Keywords:** AGEs, Myoblast, Myotube, Nox2, ROS

## Abstract

Advanced glycation end products (AGEs) are risk factors for various diseases, including sarcopenia. One of the deleterious effects of AGEs is the induction of abnormal reactive oxygen species (ROS) production in skeletal muscle. However, the underlying mechanism remains poorly understood. Therefore, the aim of this study was to elucidate how AGEs induce ROS production in skeletal muscle cells. This study demonstrated that AGEs treatment promoted ROS production in myoblasts and myotubes while PKC inhibitor abolished ROS production by AGEs stimulation. Phosphorylation of p47 phox by kinases such as PKCα is required to form the Nox2 complex, which induces ROS production. In this study, AGEs treatment promoted the phosphorylation of PKCα and p47 phox in myoblasts and myotubes. Our findings suggest that AGEs promote ROS production through the phosphorylation of PKCα and p47 phox in skeletal muscle cells.

## Introduction

Type 2 diabetes mellitus (T2DM) is a significant public health issue. Previous studies estimated that 382 million individuals had T2DM in 2013 and that this number will exceed 590 million people by 2035 worldwide [1, 2]. This indicates a rapid increase in the number of T2DM patients. T2DM is associated with mortality and morbidity, and causes multiple adverse conditions including insulin resistance and hyperglycemia [3]. Previous studies have reported that advanced glycation end products (AGEs) are accumulated in T2DM patients [4, 5]. AGEs are formed by a nonenzymatic glycation reaction known as the Maillard reaction [6], and have deleterious effects on various tissues. For instance, AGEs are recognized as risk factors for Alzheimer’s disease and sarcopenia [7, 8]. Since AGEs induce various signal pathways associated with the pathophysiology of diabetic complications [9], this study aimed to elucidate how AGEs play a deleterious role.

One of the adverse effects of AGEs is the induction of reactive oxygen species (ROS) production. Previous reports have demonstrated that AGEs induce ROS production in various cell types [10–13]. In particular, it is suggested that AGEs promote ROS production via NADPH oxidase 2 (Nox2) activation in renal glomerular tissue and hepatic stellate cells [13, 14]. The Nox2 complex is composed of gp91 phox, p22 phox, p67 phox, p47 phox, p40 phox and Rac. To activate Nox2, p47 phox is required to be phosphorylated. Protein kinase C (PKC) is phosphorylated and activated by various factors, including growth factors and hyperglycemia [15–17]. PKC phosphorylates p47 phox, activates Nox2 and induces ROS production [18].

AGEs impair the functions of skeletal muscle [19], which is the most abundant tissue in the body and has fundamental multiple functions such as movement, heat production and metabolism [20, 21]. The dysfunction and atrophy of skeletal muscle can cause several detrimental conditions and shorter life expectancy [22]. AGEs cause insulin resistance by blunting the insulin sensitivity of skeletal muscle [23], and promote ROS production in skeletal muscle tissue [24]. Excessive ROS levels impair skeletal muscle function and damage the skeletal muscle cells [25], necessitating the appropriate neutralization of ROS by antioxidant enzymes such as catalase.

While previous report has demonstrated that AGEs promote ROS production in skeletal muscle tissue [24], the signal pathway of ROS production by AGEs remains to be elucidated. In hepatic stellate cells, AGEs induce ROS production via Nox2, which is activated by PKC [13]. Therefore, we hypothesized that AGEs activate PKC and Nox2, and then promote ROS production in skeletal muscle cells.

## Materials and methods

### Cell culture

Mouse myoblast C2C12 cells were cultured in growth medium containing Dulbecco’s modified Eagle’s medium (DMEM; Thermo Fisher Scientific, Waltham, MA, USA) supplemented with 10% heat-inactivated fetal bovine serum (FBS; Biowest, Nuaillé, France) on 35 mm culture dishes (Asone, Osaka, Japan), 96 well clear bottom plates (TPP Techno Plastic Products AG, Trasadingen, Switzerland) or 96 well black bottom plates (Sumitomo Bakelite, Tokyo, Japan), which are coated with type I collagen (Corning Incorporated, Corning, NY, USA) at 37 °C in a 5% CO_2_ incubator. To differentiate C2C12 myoblasts into myotubes, the culture medium was changed to differential medium containing DMEM supplemented with 2% heat-inactivated horse serum (HS; Thermo Fisher Scientific) 2 days after myoblasts were cultured with growth medium. In this study, we used myotubes 6 days after differentiation.

### AGEs stimulation

AGEs were prepared as described previously [26]. Briefly, 50 mg/mL bovine serum albumin (BSA; Nacalai Tesque, Kyoto, Japan) was incubated with 0.1 M glyceraldehyde (Sigma-Aldrich Co., St. Louis, MO, USA) in 0.2 M phosphate buffer (pH, 7.4) under sterile conditions at 37 °C for 7 days. Unincorporated glyceraldehyde was removed by dialysis. Non-glycated BSA was incubated under the same conditions except in the absence of glyceraldehyde as a negative control.

Myoblasts were treated with 1 mg/ml AGEs or 1 mg/ml BSA for 1 h while myotubes were treated with 1 mg/ml AGEs or 1 mg/ml BSA for 24 h. To inhibit PKC activity, both myoblasts and myotubes were incubated with 100 nM PKC antagonist Go6983 (Abcam, Cambridge, UK) for 30 min prior to AGEs stimulation. These cells were used for subsequent experiments.

### ROS production analysis

To analyze ROS production, the Highly Sensitive DCFH-DA ROS Assay kit (Dojindo Laboratories, Kumamoto, Japan) was used according to the manufacture’s instruction. Briefly, cells were washed with Hanks' Balanced Salts Solution (HBSS; Thermo Fisher Scientific) after AGEs stimulation, and then incubated with DCFH-DA solution for 30 min. After washing with HBSS, ROS production was analyzed by microscopy or plate reader. For microscopic observation, images were captured at × 10 magnification using BZ-X800 (Keyence, Osaka, Japan). To analyze fluorescent intensity, Spark (Tecan, Männedorf, Switzerland) was used at excitation/emission: 490/540 nm.

### Western blotting

After AGEs stimulation, cells were lysed with radioimmunoprecipitation assay buffer (RIPA buffer) containing with 50 mM Tris, 3 mM MgCl_2_, 100 mM NaCl, 1 mM dithiothreitol (DTT), 1% triton X-100, 0.1% sodium dodecyl sulfate (SDS), 0.1% sodium deoxycholate, 1 mM phenylmethylsulfonyl fluoride (PMSF), 10 μM leupeptin, 10 mM NaF and 10 mM b-glycerophosphate. After centrifugation at 17,000 × *g* for 10 min at 4 °C, the supernatant was diluted with an equal volume of 2 × Laemmli sample buffer containing with 4% SDS, 200 mM DTT, 100 mM Tris, 20% glycerol and 0.05% bromophenol blue, and then boiled at 95 °C for 5 min. The samples were subjected to SDS-PAGE and transferred to Immobilon-P Transfer Membrane (Merck, Darmstadt, Germany). The membrane was blocked with PVDF blocking reagent (Toyobo, Osaka, Japan) for 1 h at room temperature and then incubated with anti-phospho-PKCα antibody (1:1000, sc-377565, Santa Cruz Biotechnology, Dallas, Texas, USA) diluted with TBST or anti-phospho-p47 phox antibody (1:5000, SAB4504289, Sigma-Aldrich Co.) diluted with Can Get Signal Solution 1 (Toyobo) overnight at 4 °C. Then, the membrane was incubated with anti-mouse IgG conjugated with horseradish peroxidase (HRP) antibody (1:5000, NA931V, Cytiva, Tokyo, Japan) or anti-rabbit IgG conjugated with HRP antibody (1:5000, NA934V, Cytiva) diluted with Can Get Signal Solution 2 (Toyobo). The membrane was treated with Chemi-Lumi One Ultra (Nacalai Tesque) and captured by LuminoGraph (Atto, Tokyo, Japan). To reprobe antibodies, the membrane was incubated with 15% H_2_O_2_ for 30 min at room temperature or WB Stripping Solution Strong (Nacalai Tesque) for 10 min at room temperature. Similarly, the membrane was blocked and incubated with anti-PKCα antibody (1:1000, sc-8393, Santa Cruz Biotechnology) diluted with TBST or anti-p47 phox antibody (1:1000, sc-17845, Santa Cruz Biotechnology) diluted with Can Get Signal Solution 1, incubated with appropriate secondary antibodies and then treated with Chemi-Limi One Ultra. After detection with LuminoGraph, the membrane was stained with Coomassie Brilliant Blue (CBB) solution containing with 40% MeOH, 10% AcOH and 0.05% CBB R-250 (Nacalai Tesque). Signal intensity was measured by ImageJ (National Institute of Health, Bethesda, MD, USA). The signal intensity of the target protein was normalized to total protein (CBB staining intensity).

### Statistics

All values were presented as means ± standard error of the mean (SEM). Statistical analyses were performed using GraphPad Prism 10 (GraphPad Software, Boston, MA, USA). To compare the means between the two groups, Mann–Whitney *U* test was used. Two-way ANOVA with Tukey’s multiple comparison test was employed for comparisons of the mean among the four groups. Differences among groups were considered significant at *p* < 0.05.

## Results

### AGEs promote ROS production in myoblasts

**Fig. 1:**
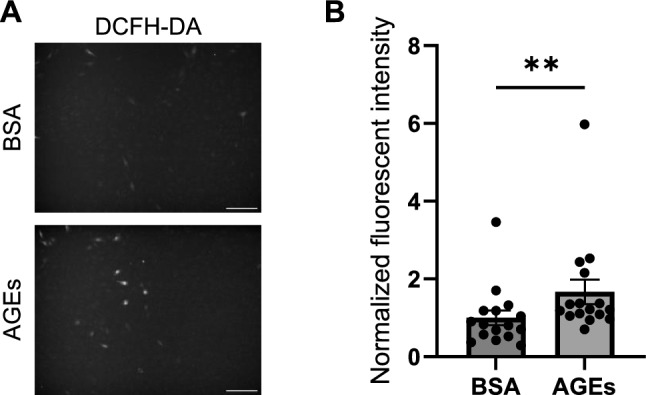
Advanced glycation end products (AGEs) promote radical oxygen species (ROS) production in C2C12 myoblasts. Myoblasts are treated with 1 mg/ml AGEs or 1 mg/ml BSA for 1 h. **A** The representative image of myoblasts stained with DCFH-DA. Scale bars, 200 μm. **B** Quantification of fluorescent intensity of myoblasts stained with DCFH-DA (n = 16). Error bars show mean ± SEM. ** indicates *p* < 0.01 by Mann–Whitney *U* test

To examine the effect of AGEs on ROS production in myoblasts, C2C12 cells were treated with 1 mg/ml AGEs or 1 mg/ml BSA for 1 h. BSA was used as control for AGEs. After the cells were treated with DCFH-DH, ROS production was measured. Consequently, AGEs treatment significantly increased the fluorescent intensity compared to BSA treatment, suggesting that AGEs promote ROS production in myoblasts (Fig. 1).

### AGEs induce phosphorylation of PKCα and p47 phox in myoblasts

Because previous studies reported that PKCα induces phosphorylation of p47 phox, activates Nox2, and then promotes ROS production [14, 18, 27], we hypothesized that AGEs induce the phosphorylation of PKCα and p47 phox, and then promote ROS production in myoblasts.

**Fig. 2:**
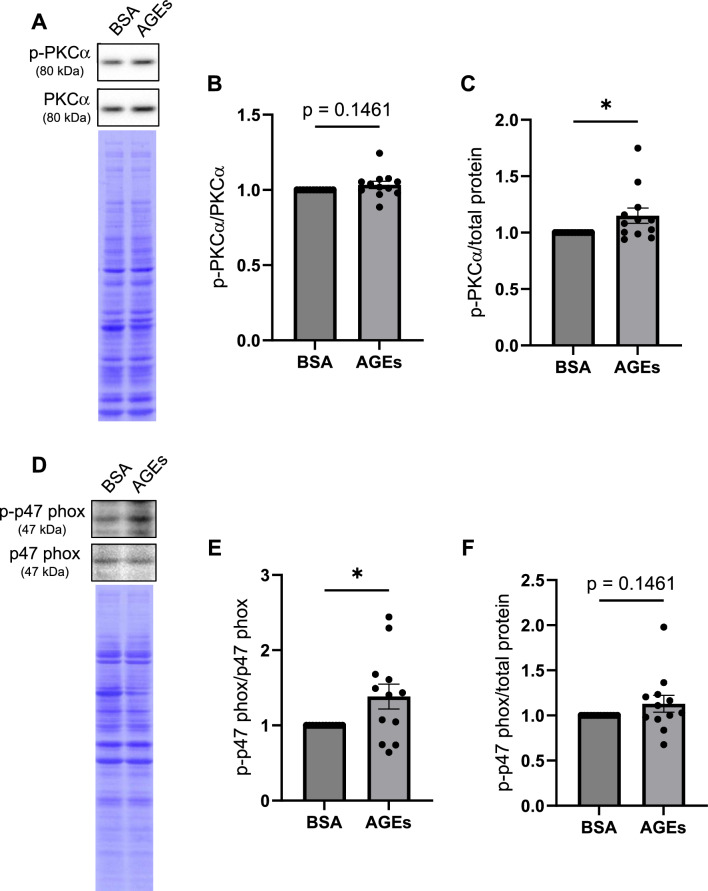
AGEs promote phosphorylation of PKCα and p47 phox in C2C12 myoblasts. Myoblasts are treated with 1 mg/ml AGEs or 1 mg/ml BSA for 1 h. **A** The representative immunoblot image of p-PKCα and PKCα. **B**–**C** Quantification of the expression level of p-PKCα normalized to PKCα or total protein (n = 12). **D** The representative immunoblot image of p-p47 phox and p47 phox. **E**–**F** Quantification of the expression level of p-p47 phox normalized to p47 phox or total protein (n = 12). For all graphs, error bars show mean ± SEM. * indicates *p* < 0.05 by Mann–Whitney *U* test

To verify this hypothesis, myoblasts were stimulated with AGEs as described above, and the amount of p-PKCα and p-p47 phox was measured. While the ratio of p-PKCα to PKCα was tended to be increased, p-PKCα was significantly increased by AGEs stimulation (Fig. 2A–C). While the ratio of p-p47 phox to p47 phox was significantly increased, p-p47 phox was tended to be increased by AGEs treatment (Fig. 2D–F). Taken together, it is suggested that AGEs promote the phosphorylation of both PKCα and p47 phox in myoblasts.

### ROS production is induced by AGEs in myotubes

**Fig. 3:**
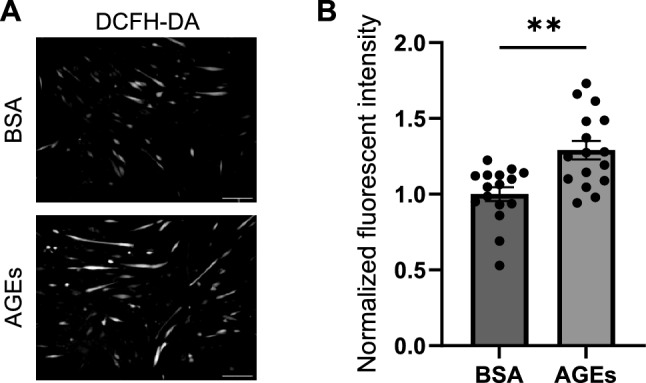
AGEs promote ROS production in C2C12 myotubes. Myotubes are treated with 1 mg/ml AGEs or 1 mg/ml BSA for 24 h. **A** The representative image of myotubes stained with DCFH-DA. Scale bars, 200 μm. **B** Quantification of fluorescent intensity of myotubes stained with DCFH-DA (n = 16). Error bars show mean ± SEM. ** indicates *p* < 0.01 by Mann–Whitney *U* test

To analyze the effect of AGEs on ROS induction in myotubes, myotubes were treated with 1 mg/ml AGEs or 1 mg/ml BSA for 24 h. After DCFH-DA treatment, the fluorescent intensity was significantly increased by AGEs treatment, indicating that AGEs treatment promotes ROS production in myotubes (Fig. 3).

### Phosphorylation of PKCα and p47 phox is promoted by AGEs in myotubes

**Fig. 4:**
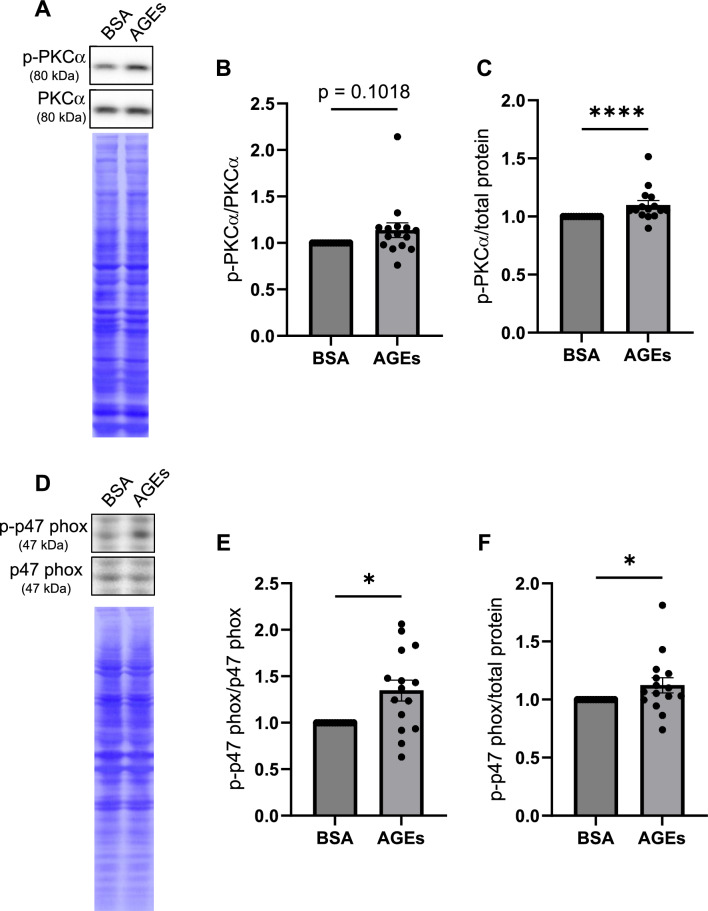
AGEs promote phosphorylation of PKCα and p47 phox in C2C12 myotubes. Myotubes are treated with 1 mg/ml AGEs or 1 mg/ml BSA for 24 h. **A** The representative immunoblot image of p-PKCα and PKCα. **B**–**C** Quantification of the expression level of p-PKCα normalized to PKCα or total protein (n = 15). **D** The representative immunoblot image of p-p47 phox and p47 phox. **E**–**F** Quantification of the expression level of p-p47 phox normalized to p47 phox or total protein (n = 15). For all graphs, error bars show mean ± SEM. * and **** indicate *p* < 0.05 and *p* < 0.0001, respectively, by Mann–Whitney *U* test

To examine whether AGEs promote the phosphorylation of PKCα and p47 phox in myotubes, myotubes were treated with AGEs in a similar manner as described above. While the ratio of p-PKCα to PKCα was tended to be increased, p-PKCα was significantly increased by AGEs treatment (Fig. 4A–C). AGEs stimulation significantly increased both p-p47 phox and the ratio of p-p47 phox to p47 phox (Fig. 4D–F). Collectively, it is suggested that AGEs induce the phosphorylation of both PKCα and p47 phox in myotubes.

### PKC antagonist abolishes ROS induction by AGEs in myoblasts and myotubes

**Fig.5:**
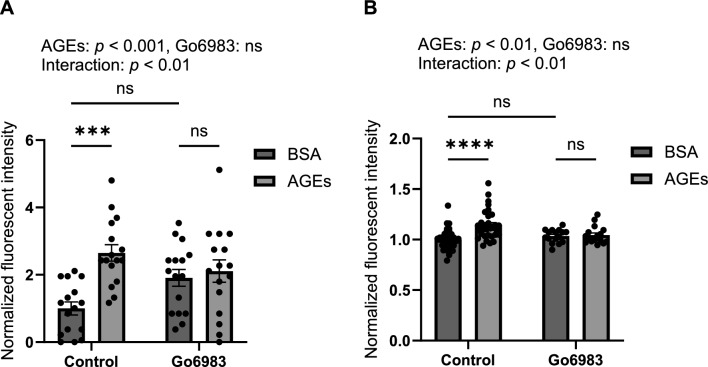
PKC inhibitor abrogates ROS induction by AGEs treatment in C2C12 myoblasts and myotubes. Following a 30 min preincubation with 100 nM Go6983, myoblasts are treated with 1 mg/ml AGEs or 1 mg/ml BSA for 1 h while myotubes are treated with 1 mg/ml AGEs or 1 mg/ml BSA for 24 h. **A** Quantification of fluorescent intensity of myoblasts (n = 16) and **B** myotubes stained with DCFH-DA (n = 16–32). For all graphs, error bars show mean ± SEM. ns, *** and **** indicate *p* > 0.05, *p* < 0.001 and *p* < 0.0001, respectively, by two-way ANOVA with Tuker’s multiple comparison test

To confirm whether AGEs promote ROS production via PKC, cells were treated with 100 nM PKC antagonist Go6983 for 30 min prior to AGEs stimulation. AGEs stimulation induced ROS production in both myoblasts and myotubes whereas Go6983 abolished ROS induction by AGEs, suggesting that AGEs induce ROS production via PKC (Fig. 5).

## Discussion

This study investigated how AGEs induce ROS production in skeletal muscle cells. We showed that AGEs treatment increased the fluorescent intensity of DCFH-DA staining in myoblasts and myotubes, suggesting that AGEs promote ROS production (Figs. 1, 3). AGEs treatment also promoted the phosphorylation of PKCα and p47 phox in myoblasts and myotubes (Figs. 2, 4). Considering that PKC inhibitor abolished ROS production by AGEs stimulation (Fig. 5), we propose that AGEs induce ROS production through PKC/Nox2 axis in skeletal muscle.

In this study, AGEs promoted ROS production in myoblasts and myotubes in vitro (Figs. 1, 3). A previous report also suggests that ROS production is increased by AGEs treatment in skeletal muscle tissue in vivo [24]. However, the mechanism of ROS induction by AGEs in skeletal muscle is not fully understood. Because AGEs promote ROS production via Nox2 in hepatic stellate cells [13], we speculated that ROS production is induced by AGEs through Nox2. In this study, AGEs treatment promoted the phosphorylation of p47 phox, a component of the Nox2 complex, in myoblasts and myotubes (Figs. 2D–F, 4D–F). To produce ROS via Nox2, it is necessary to form the complex consisting of gp91 phox, p22 phox, p67 phox, p47 phox, p40 phox and Rac [18]. All components of the Nox2 complex are expressed in myoblasts and myotubes [28, 29]. Phosphorylation of p47 phox is required for the Nox2 complex formation. Considering that AGEs induce the phosphorylation of p47 phox (Figs. 2D–F, 4D–F), it is plausible that AGEs promote the ROS production via the phosphorylation of p47 phox in myoblasts and myotubes.

PKC is one of the kinases which phosphorylate p47 phox [27]. PKC belongs to a family of serine/threonine kinases and regulates a myriad of fundamental signal pathways, leading to the control of cell growth, differentiation, apoptosis and so on [30]. PKC is phosphorylated and activated by various ligands such as growth factors and Wnt [15, 31]. Since a previous report demonstrated that AGEs induce the phosphorylation of PKCα and ROS production in renal glomerular tissue [14], we assumed that AGEs also transduce the same signal pathway in skeletal muscle. Indeed, AGEs treatment promoted the phosphorylation of PKCα in myoblasts and myotubes (Figs. 2A–C, 4A–C). Taken together, this study suggests that PKCα is phosphorylated by AGEs treatment, and then PKCα phosphorylates p47 phox.

T2DM is a metabolic disorder which develops multifactorial symptoms including hyperglycemia. Hyperglycemia accelerates the formation of AGEs, which are involved in the pathogenesis of T2DM and diabetic complications [4, 9]. T2DM and AGEs cause multiple deleterious conditions such as abnormal ROS production [10–12, 32]. Excessive ROS burden impairs skeletal muscle function and causes frailty [33]. Considering that this study demonstrated that AGEs induce ROS production via Nox2 in skeletal muscle cells, Nox2 inhibitors may protect skeletal muscle from excessive ROS production. Indeed, gp91ds-tst, a peptide inhibitor of Nox2, mitigates skeletal muscle atrophy induced by hindlimb unloading [34].

The primary receptor of AGEs is the receptor for advanced glycation end products (RAGE). The interaction between AGEs and RAGE transduces various signal pathways, resulting in pathological conditions. Previous reports have demonstrated that AGE/RAGE signal activates PKC in cardiomyocytes [35] and induces ROS production via Nox2 in human umbilical vein endothelial cells (HUVEC) [11]. Together with this study, it is possible that AGEs interact with RAGE, phosphorylate PKC and p47 phox, and then induce ROS production.

We acknowledge that this study has several limitations. First, although this study demonstrated that AGEs promote the phosphorylation of PKCα and p47 phox in skeletal muscle cells (Figs. 2, 4), previous studies have suggested that AGEs induce ROS production by different PKC isoforms [10, 13, 14, 16] and these PKC isoforms phosphorylate p47 phox [27]. Therefore, it is possible that AGEs may phosphorylate p47 phox via activation of not only PKCα but also other PKC isoforms. Second, AGEs may promote ROS production via mitochondrial dysfunction as well as Nox2 activation in skeletal muscle cells. In Achilles tendon-derived fibroblasts, AGEs impair mitochondrial function [36]. Mitochondrial dysfunction causes abnormal ROS production [37]. Thus, further studies are needed to elucidate whether AGEs phosphorylate other PKC isoforms and whether AGEs induce ROS production via mitochondrial dysfunction.

Our results demonstrated that AGEs promoted ROS production in myoblasts and myotubes (Figs. 1, 3) while PKC inhibitor abolished ROS induction by AGEs (Fig. 5). p47 phox is a component of the Nox2 complex, which is one of the molecular systems associated with the induction of ROS production. AGEs promoted the phosphorylation of PKCα and p47 phox in myoblasts and myotubes (Figs. 2, 4). Therefore, this study suggests that AGEs-induced ROS production is mediated by PKC/p47 phox axis.

## Data Availability

The data underpinning the findings of this study are available from the corresponding author, TE, upon reasonable request.
